# Supplementary material for: End Sequence Analysis Toolkit (ESAT) expands the extractable information from single-cell RNA-seq data
Source: Genome Res. 2016 Oct;26(10):1397–410. doi: 10.1101/gr.207902.116 (PMC5052061; doi:10.1101/gr.207902.116)
Supplement: Supplemental Material [file supp_gr.207902.116_Supp_Materials.tar.gz › ESAT-esat_v0.1/doc/html/classbroad_1_1core_1_1util_1_1_collapse_by_intersection.html]

ESAT: broad.core.util.CollapseByIntersection Class Reference


ESAT


- Main Page
- Related Pages
- Packages
- Classes
- Files

- Class List
- Class Index
- Class Hierarchy
- Class Members

All Classes Namespaces Files Functions Variables Enumerator Pages

- broad
- core
- util
- CollapseByIntersection

Static Public Member Functions |
List of all members

broad.core.util.CollapseByIntersection Class Reference

|  |  |
| --- | --- |
| Static Public Member Functions | |
| static Set< Annotation > | collapseByIntersection (Collection<?extends Annotation > alignments, boolean intersection) |
|  | |
| static Collection< Annotation > | DecollapseByIntronLocation (Collection< Annotation > exons, Collection< Annotation > introns, String strand) |
|  | |
| static Collection< Annotation > | filter (Collection< Annotation > introns, String strand) |
|  | |
| static Collection< Annotation > | DecollapseByIntronLocation (Collection< Annotation > exons, Collection< Annotation > introns) |
|  | |
| static Collection< Annotation > | DecollapseByIntronLocationOld (Collection< Annotation > exons, Collection< Annotation > introns) |
|  | |
| static Collection< Annotation > | DecollapseByIntronLocationWorking (Collection< Annotation > exons, Collection< Annotation > introns) |
|  | |
| static Collection< Gene > | updateBoundariesWithoutCrossingIntrons (Collection< Gene > genes) |
|  | |
| static Map< String,   IntervalTree< Annotation > > | makeIntervalTree (Collection<?extends Annotation > alignments) |
|  | |
| static Map< String,   IntervalTree< Gene > > | makeIntervalTreeForGenes (Collection< Gene > alignments) |
|  | |
| static Collection< Annotation > | CollapseGenesByIntersection (Collection< Gene > temp, boolean intersection) |
|  | |
| static Map< String,   IntervalTree< Annotation > > | makeIntervalTreeForGeneExons (Collection< Gene > genes) |
|  | |

## Member Function Documentation

|  |  |  |  |
| --- | --- | --- | --- |
| static Set<Annotation> broad.core.util.CollapseByIntersection.collapseByIntersection | ( | Collection<?extends Annotation > | *alignments*, |
|  |  | boolean | *intersection* |
|  | ) |  |  |

static

Here is the call graph for this function:

Here is the caller graph for this function:

|  |  |  |  |
| --- | --- | --- | --- |
| static Collection<Annotation> broad.core.util.CollapseByIntersection.CollapseGenesByIntersection | ( | Collection< Gene > | *temp*, |
|  |  | boolean | *intersection* |
|  | ) |  |  |

static

Here is the call graph for this function:

|  |  |  |  |
| --- | --- | --- | --- |
| static Collection<Annotation> broad.core.util.CollapseByIntersection.DecollapseByIntronLocation | ( | Collection< Annotation > | *exons*, |
|  |  | Collection< Annotation > | *introns*, |
|  |  | String | *strand* |
|  | ) |  |  |

static

This method returns only exons that are trimmed down by intron location. If its fully within intron it just returns it. If bases overlap intron it trims down

Parameters
:   |  |  |
    | --- | --- |
    | exons |  |
    | introns |  |
    | strand |  |

Returns

Here is the call graph for this function:

Here is the caller graph for this function:

|  |  |  |  |
| --- | --- | --- | --- |
| static Collection<Annotation> broad.core.util.CollapseByIntersection.DecollapseByIntronLocation | ( | Collection< Annotation > | *exons*, |
|  |  | Collection< Annotation > | *introns* |
|  | ) |  |  |

static

Here is the call graph for this function:

|  |  |  |  |
| --- | --- | --- | --- |
| static Collection<Annotation> broad.core.util.CollapseByIntersection.DecollapseByIntronLocationOld | ( | Collection< Annotation > | *exons*, |
|  |  | Collection< Annotation > | *introns* |
|  | ) |  |  |

static

Here is the call graph for this function:

|  |  |  |  |
| --- | --- | --- | --- |
| static Collection<Annotation> broad.core.util.CollapseByIntersection.DecollapseByIntronLocationWorking | ( | Collection< Annotation > | *exons*, |
|  |  | Collection< Annotation > | *introns* |
|  | ) |  |  |

static

Here is the call graph for this function:

|  |  |  |  |
| --- | --- | --- | --- |
| static Collection<Annotation> broad.core.util.CollapseByIntersection.filter | ( | Collection< Annotation > | *introns*, |
|  |  | String | *strand* |
|  | ) |  |  |

static

Here is the caller graph for this function:

|  |  |  |  |  |  |
| --- | --- | --- | --- | --- | --- |
| static Map<String, IntervalTree<Annotation> > broad.core.util.CollapseByIntersection.makeIntervalTree | ( | Collection<?extends Annotation > | *alignments* | ) |  |

static

Here is the caller graph for this function:

|  |  |  |  |  |  |
| --- | --- | --- | --- | --- | --- |
| static Map<String, IntervalTree<Annotation> > broad.core.util.CollapseByIntersection.makeIntervalTreeForGeneExons | ( | Collection< Gene > | *genes* | ) |  |

static

Here is the call graph for this function:

|  |  |  |  |  |  |
| --- | --- | --- | --- | --- | --- |
| static Map<String, IntervalTree<Gene> > broad.core.util.CollapseByIntersection.makeIntervalTreeForGenes | ( | Collection< Gene > | *alignments* | ) |  |

static

Here is the caller graph for this function:

|  |  |  |  |  |  |
| --- | --- | --- | --- | --- | --- |
| static Collection<Gene> broad.core.util.CollapseByIntersection.updateBoundariesWithoutCrossingIntrons | ( | Collection< Gene > | *genes* | ) |  |

static

Here is the call graph for this function:

---

The documentation for this class was generated from the following file:

- java/broad/core/util/CollapseByIntersection.java


---

Generated on Fri Aug 15 2014 15:10:57 for ESAT by  

 1.8.7
